# A New Strategy for Heavy Metal Polluted Environments: A Review of Microbial Biosorbents

**DOI:** 10.3390/ijerph14010094

**Published:** 2017-01-19

**Authors:** Ayansina Segun Ayangbenro, Olubukola Oluranti Babalola

**Affiliations:** Food Security and Safety Niche Area, Faculty of Agriculture, Science and Technology, North-West University, Private Bag X2046, Mmabatho 2735, South Africa; 28072693@g.nwu.ac.za

**Keywords:** bioremediation, biosorbent, biosorption, heavy metals, microorganisms, remediation

## Abstract

Persistent heavy metal pollution poses a major threat to all life forms in the environment due to its toxic effects. These metals are very reactive at low concentrations and can accumulate in the food web, causing severe public health concerns. Remediation using conventional physical and chemical methods is uneconomical and generates large volumes of chemical waste. Bioremediation of hazardous metals has received considerable and growing interest over the years. The use of microbial biosorbents is eco-friendly and cost effective; hence, it is an efficient alternative for the remediation of heavy metal contaminated environments. Microbes have various mechanisms of metal sequestration that hold greater metal biosorption capacities. The goal of microbial biosorption is to remove and/or recover metals and metalloids from solutions, using living or dead biomass and their components. This review discusses the sources of toxic heavy metals and describes the groups of microorganisms with biosorbent potential for heavy metal removal.

## 1. Introduction

Industrialization and technological advancement have put an increasing burden on the environment by releasing large quantities of hazardous waste, heavy metals (cadmium, chromium, and lead) and metalloids (elements with intermediate properties between those of typical metals and non-metals, such as arsenic and antimony), and organic contaminants that have inflicted serious damage on the ecosystem. The build-up of heavy metals and metalloids in soils and waters continues to create serious global health concerns, as these metals and metalloids cannot be degraded into non-toxic forms, but persist in the ecosystem. Contamination of the environment with heavy metals has increased beyond the recommended limit and is detrimental to all life forms [1,2,3]. The maximum permissible concentration of some heavy metals in water, as stated by the Comprehensive Environmental Response Compensation and Liability Act (CERCLA), USA, is 0.01, 0.05, 0.01, 0.015, 0.002, and 0.05 mg/L for Ar, Cd, Cr, Pb, Hg, and Ag respectively [4]. The standard for soil, as established by the Indian standards for heavy metals, is 3–6, 135–270, 75–150, 250–500, and 300–600 mg/kg for Cd, Cu, Ni, Pb, and Zn respectively [5].

Heavy metal pollution is currently a major environmental problem because metal ions persist in the environment due to their non-degradable nature. The toxicity and bioaccumulation tendency of heavy metals in the environment is a serious threat to the health of living organisms. Unlike organic contaminants, heavy metals cannot be broken down by chemical or biological processes. Hence, they can only be transformed into less toxic species.

The majorities of heavy metals are toxic at low concentrations and are capable of entering the food chain, where they accumulate and inflict damage to living organisms. All metals have the potential to exhibit harmful effects at higher concentrations and the toxicity of each metal depends on the amount available to organisms, the absorbed dose, the route and the duration of exposure [6]. Due to the noxious effects of these metals, there are growing environmental and public health concerns, and a consequent need for increase awareness in order to remediate the heavy metal polluted environment. Thus, it is imperative to remove or reduce heavy metal contamination in order to prevent or reduce contaminating the environment and the possibility of uptake in the food web. To achieve this, bioremediation is employed in order to increase metal stability (speciation), which in turn reduces the bioavailability of metal [7,8,9]. Speciation is defined as the identification and quantification of the different, defined species, forms, or phases, in which a metal occurs, while bioavailability is the portion of the total amount of a metal in an environment, within a time frame, that is available or made available for uptake by living organisms in their direct surroundings. Speciation of metal and its bioavailability determines the physiological and toxic effects of a metal on living organisms [10].

Bioremediation is a state-of-the-art technique used for heavy metal removal and/or recovery from polluted environments. The technique utilizes inherent biological mechanisms to eradicate hazardous contaminants using microorganisms and plants, or their products, to restore polluted environments to their original condition [2,6,8]. It is an environmentally friendly and cost-effective technique for heavy metal removal/recovery, when compared to the conventional chemical and physical techniques, which are often more expensive and ineffective, especially for low metal concentrations. In addition, these conventional methods generate significant amounts of toxic sludge.

Microbial remediation is described as the use of microorganisms to perform the absorption, precipitation, oxidation, and reduction of heavy metals in the soil [11]. Microorganisms possess astonishing metabolic pathways which utilize various toxic compounds as a source of energy for growth and development, through respiration, fermentation, and cometabolism. Due to their characteristic degradative enzymes for a particular contaminant, they have evolved diverse mechanisms for maintaining homeostasis and resistance to heavy metals, in order to adapt to toxic metals in the ecosystem [12,13]. Strategies developed by microorganisms for continued existence in heavy metal polluted environments, include mechanisms such as bioaccumulation, biomineralization, biosorption, and biotransformation. These mechanisms are exploited for in situ (treatment at the site of contamination), or ex situ (the contaminated site can be excavated or pumped and treated away from the point of contamination), remediation. Owing to these abilities, they have been effectively used as biosorbents for heavy metal removal and recovery. The majority of heavy metals disrupt microbial cell membranes, but microorganisms can develop defense mechanisms that assist them in overcoming the toxic effect. Thus, the response of microorganisms to heavy metal toxicity is of importance for re-establishing polluted sites.

This article presents insights into the use of microbial biosorbents for removing heavy metals from industrial waste and contaminated environments, as well as the sources and toxicity of these metals in the food web.

## 2. Sources of Heavy Metal Pollution in the Environment

Naturally occurring heavy metals are present in forms that are not readily available for uptake by plants. They are typically present in insoluble forms, like in mineral structures, or in precipitated or complex forms that are not readily available for plant uptake. Naturally occurring heavy metals have a great adsorption capacity in soil and are thus not readily available for living organisms. The bonding energy between naturally occurring heavy metals and soil is very high compared to that with anthropogenic sources. Examples of natural processes that bring about the occurrence of heavy metals in the environment are comets, erosion, volcanic eruptions, and the weathering of minerals. Heavy metals from anthropogenic sources typically have a high bioavailability due to their soluble and mobile reactive forms. These anthropogenic sources include alloy production, atmospheric deposition, battery production, biosolids, coating, explosive manufacturing, improper stacking of industrial solid waste, leather tanning, mining, pesticides, phosphate fertilizer, photographic materials, printing pigments, sewage irrigation, smelting, steel and electroplating industries, textiles, and dyes and wood preservation [2,14] (Table 1). Sources of heavy metals, concentrations in soil, soil properties, the degree and extent of uptake by plants, and the extent of absorption by animals, are the factors that influence the accumulation of metal ions in the food web [15]. According to D’amore, et al. [16], the geochemical cycle of heavy metals results in the buildup of heavy metals in the environment, which could cause risk to all life forms when they are above permitted levels. The routes of entry into the environment usually include the weathering of parent materials, the alteration of the geochemical cycle by man, soil ingestion (which is the primary exposure route to humans of soil-borne metals), the transfer from mines to other locations, and the discharge of high concentrations of metal waste by industries.

Mining has negatively impacted the environment, causing destruction and an alteration of the ecosystem, including a loss of biodiversity and an accumulation of pollutants in the environment. Mining and ore processing are major sources of heavy metal pollution in the soil, and the recovery of ecosystems from mining activities could take several decades. These activities produced large quantities of stockpiles and dumps, which are frequently abandoned without treatment. Abandoned mines contaminate water bodies through chemical run-off and particulates that accumulate in water sources [17], hence, creating a need to treat wastewaters contaminated with heavy metals, before discharge into the environment occurs.

## 3. Toxicity of Heavy Metals to Life Forms

Although some heavy metals play important roles in the physiological, biochemical, and metabolic processes of living organisms, functioning as co-factors for some enzymes, micronutrients, regulators of osmotic pressure, and stabilization of molecules, the majority of them have no known biological function in living organisms and are toxic when generated in excess [24]. The toxicity of metals is the ability of a metal to cause undesirable effects on organisms. This depends on the heavy metal bioavailability and the absorbed dose [42]. The threat posed by heavy metals to the health of living organisms is worsened by their continuously persistent nature in the environment. Toxicity increases when the medium becomes acidic and nutrient-deficient, and when the soil structure is poor, especially in mining environments [43].

At acidic pH levels, heavy metals tend to form free ionic species, with more protons available to saturate metal binding sites. This means that at higher hydrogen ion concentrations, the adsorbent surface is further positively charged, thus reducing the attraction between adsorbent and metal cations. Therefore, heavy metal becomes more bioavailable, thereby increasing its toxicity to microorganisms and plants. At basic conditions, metal ions replace protons to form other species, such as hydroxo-metal complexes. These complexes are soluble in some cases (Cd, Ni, Zn), while those of Cr and Fe are insoluble. The solubility and bioavailability of heavy metals can be influenced by a small change in the pH level. Variations in soil composition, such as the organic matter content of a soil, also affect the toxicity of heavy metals. In soil with relatively low organic matter content, high contamination by heavy metals is usually observed. Organic matter content has a strong influence on the cation exchange capacity, buffer capacity, as well as on the retention of heavy metals. Thus, metals present in organic soils contaminated with a combination of heavy metals are less mobile and less bioavailable to microorganisms and plants, than metals present in mineral soils [10].

Temperature also plays an important role in the adsorption of heavy metals. It has two major effects on the adsorption process. Increasing the temperature will also increase the rate of adsorbate diffusion across the external boundary layer and in the internal pores of the adsorbate particles, because liquid viscosity decreases as temperature increases. It also affects the equilibrium capacity of the adsorbate, depending on whether the process is exothermic or endothermic. Temperature changes affect the stability of the metal ion species initially placed in solution; stability of the microorganism–metal complex depends on the biosorption sites, microbial cell wall configuration, and ionization of chemical moieties on the cell wall. An increase in the sorption capacity of lead, from 0.596 to 0.728 mg/g, was obtained when the temperature was raised from 25 to 40 °C [44].

Metal toxicity is also shown in their ability to disrupt enzyme structures and functions by binding with thiol and protein groups, or by replacing co-factors in prosthetic groups of enzymes. Exposure to lead and mercury can cause the development of autoimmunity, which can result in joint diseases, such as rheumatoid arthritis, kidney diseases, circulatory and nervous system disorders, and the damaging of the fetal brain in humans. Exposure to lead and mercury in children causes reduced intelligence, impaired development, and an increased risk of cardiovascular disease. Cadmium is known to be carcinogenic and mutagenic, and can disrupt the endocrine system, damage fragile bones and lungs, and affect the regulation of calcium in biological systems. Chromium causes hair loss, headaches, diarrhea, nausea, and vomiting in humans (Table 1).

Heavy metal contaminated soils limit plant habitats due to toxicity, resulting in ecological, evolutionary, and nutritional problems, as well as severe selection pressures [6,20]. The toxicity of heavy metals in plants varies, depending on the plant species, specific metal involved, concentration of metal, chemical form of metal, and soil composition and pH [5]. There can be a build-up of heavy metals in plant tissues that affects or inhibits nutrient uptake, homoeostasis, growth, and development. They disrupt metabolic functions, such as physiological and biochemical processes, biochemical lesions, cell organelles destruction, chlorosis, delayed germination, induced genotoxicity, inhibition of photosynthesis and respiration, loss of enzyme activities, oxidative stress, premature leaf fall, reduced biomass, reduced crop yield, senescence, stunted growth, wilting and can even cause the death of plants (Table 1).

Heavy metal toxicity affects microbial population size, diversity, and activity, as well as their genetic structure. It affects the morphology, metabolism, and growth of microorganisms by altering the nucleic acid structure, disrupting the cell membranes, causing functional disturbance, inhibiting enzyme activity and oxidative phosphorylation, and causing lipid peroxidation, osmotic balance alteration, and protein denaturation [24,45] (Table 1).

## 4. Bioremediation of Heavy Metals by Microorganisms

Several techniques have been used for the removal and/or recovery of heavy metals from polluted environments. Some established conventional procedures for heavy metal removal and/or recovery from solution, include adsorption processes, chemical oxidation or reduction reactions, chemical precipitation, electrochemical techniques, evaporative recovery, ion exchange, reverse osmosis, and sludge filtration [46]. However, these techniques are expensive, sometimes impracticable, and are not specific for metal-binding properties. Furthermore, the generation of toxic waste, the high reagent requirement, and the unpredictable nature of metal ion removal, highlights some of the disadvantages of these methods. The majority of these methods are ineffective when metal concentrations in solution are less than 100 mg/L [47]. Separation by physical and chemical techniques is also challenging due to the high solubility of most heavy metal salts in solution. Thus, there is a need to evaluate alternative techniques for a given procedure and such an approach should be suitable, appropriate, and applicable to the local conditions, and must be able to meet the established permissible limits.

Bioremediation is an innovative technique for the removal and recovery of heavy metal ions from polluted areas, and involves using living organisms to reduce and/or recover heavy metal pollutants into less hazardous forms, using the activities of algae, bacteria, fungi, or plants. It has been employed for the removal of heavy metals from contaminated wastewaters and soils. This method is an appealing alternative to physical and chemical techniques, and the use of microorganisms play a significant role in heavy metal remediation. Similarly, the use of microorganisms to remediate polluted environments is sustainable and helps to restore the natural state of the polluted environment with long term environmental benefits and cost effectiveness [2]. These organisms help to detoxify hazardous components in the environment. The process can function naturally or can be improved through the addition of electron acceptors, nutrients, or other factors.

Detoxification can occur through the valence transformation mechanism. This is particularly applicable in the case of metals whose different valence states vary in toxicity. In mercury-resistant bacteria, organomercurial lyase converts methyl mercury to Hg(II), which is one hundred-fold less toxic than methyl mercury [48]. The reduction of Cr(VI) to Cr(III) is widely studied, with Cr(III) having less mobility and toxicity. Other detoxification mechanisms of heavy metals are accomplished through metal binding, vacuole compartmentalization, and volatilization. Metal binding involves chelators, such as metallothein, glutathione-derived-peptides called phytochelatin, and metal binding peptides. These chelators bind to heavy metals and facilitate microbial absorption and the transportation of metal ions. Volatilization mechanisms involve turning metal ions into a volatile state. This is only possible with Se and Hg, which have volatile states. Mercury-resistant bacteria utilizes the MerA enzyme to reduce Hg(II) to the volatile form Hg(0) [48]. The reduction of Se(V) to elemental Se(0) has been employed to remediate contaminated waters and soils. The metabolic processes of these organisms help to transform pollutants in the environment [46].

Biosorption, bioaccumulation, biotransformation, and biomineralization are the techniques employed by microorganisms for their continued existence in metal polluted environment. These strategies have been exploited for remediation procedures [49,50]. Heavy metal removal can be carried out by living organisms or dead biological materials. Large scale feasibility applications of biosorptive processes have shown that dead biomass is more applicable than the bioaccumulation approach, which involves the use of living organisms and thus requires nutrient supply and a complicated bioreactor system. Also, the toxicity of pollutants, as well as other unfavorable environmental conditions, can contribute to the inability to maintain a healthy microbial population. However, many characteristic attributes of living microorganisms have not been exploited in large scale applications [51]. The choice organism must develop resistance towards metal ions as it comes into contact with the heavy metal pollutant to achieve the goal of remediation. The organism of choice may be native to the polluted environment, or isolated from another environment and brought to the contaminated site [52].

Advances in the understanding of metabolic pathways of microorganisms are responsible for metal sequestration, improving microbial survival rates, and their stability. This has led to the manipulation of metal adsorption [53]. Adsorption is the physical adherence of ions and molecules onto the surface of another molecule. The material accumulated at the interface is the adsorbate and the solid surface is the adsorbent. If adsorption occurs and results in the formation of a stable molecular phase at the interface, this can be described as a surface complex. Most solids, including microorganisms, possess functional groups like –SH, –OH, and –COOH on their surfaces, that helps in the adsorption of metals [54]. It has been reported that a microbial cell develops resistance to heavy metals through the excretion of metal chelating substances, or through a problem in a particular transport system, which results in a reduced cell accumulation of the metal ion. Another resistance mechanism includes the binding of a metal ion to intracellular molecules, such as metallothionein, vacuole, or mitochondria, which results in changes in the distribution of metal ion [46]. Microorganisms interact with metal ions through cell wall associated metals, intracellular accumulation, metalsiderophore, extracellular polymeric reactions with transformation, extracellular mobilization or immobilization of metal ions, and volatilization of metals [46].

Various factors influence the microbial remediation of metals. They include the bioavailability of the metal to the microbe, concentration of pollutants, electron acceptors, moisture content, nutrients, osmotic pressure, oxygen, pH, redox potential, soil structure, temperature, and water activity. The bioavailability of each metal in soil is influenced by factors such as the buffering capacity, cation exchange capacity, clay minerals content, metal oxide, and organic matter [3,6,12]. In general, remediation of heavy metal is achieved through the removal of the metal ion from substratum to reduce the risk posed by exposure to such heavy metals.

The environmental conditions, prehistory, and pretreatment required for the removal of heavy metals need to be established in order to select the most appropriate biosorbent for a specific situation, from the extremely large pool of organisms that are readily available. Sometimes, the interest may be to recover a specific metal regardless of equilibrium concentration attained, or on the other hand, the interest may be to curtail levels of pollution in the effluent, in order to fall within the acceptable containment limit. Also, priority may be given to the recovery of a large quantity of metal, while also achieving low equilibrium concentrations. Whatever the case, the biosorbent used should have a high sorption capacity [55].

## 5. Mechanisms of Heavy Metal Uptake by Microorganisms

**Figure 1:**
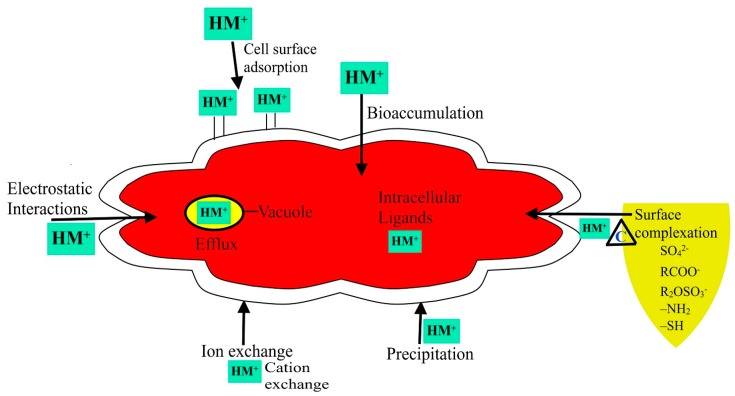
Mechanisms of heavy metal uptake by microorganisms.

The cellular structure of a microorganism can trap heavy metal ions and subsequently sorb them onto the binding sites of the cell wall [36]. This process is called biosorption or passive uptake, and is independent of the metabolic cycle. The amount of metal sorbed depends on the kinetic equilibrium and composition of the metal at the cellular surface. The mechanism involves several processes, including electrostatic interaction, ion exchange, precipitation, the redox process, and surface complexation [56] (Figure 1). The process is fast and can reach equilibrium within a few minutes. Biosorption can be carried out by fragments of cells and tissues, or by dead biomass or living cells as passive uptake via surface complexation onto the cell wall and other outer layers [57]. The other method is a process in which the heavy metal ions pass across the cell membrane into the cytoplasm, through the cell metabolic cycle. This is referred to as bioaccumulation or active uptake. Bioaccumulation is a process of a living cell that is dependent on a variety of physical, chemical, and biological mechanisms (Figure 1). These factors include intracellular and extracellular processes, where biosorption plays a limited and ill-defined role [57]. The organism that will accumulate heavy metals should have a tolerance to one or more metals at higher concentrations, and must exhibit enhanced transformational abilities, changing toxic chemicals to harmless forms that allows the organism to lessen the toxic effect of the metal, and at the same time, keep the metal contained [58].

Metal uptake mechanisms by various biosorbents depend on the cellular surface of the microbes, as well as the exchange of metal ions and complex formations with the metal ions on the reactive chemical sites of the cell surface. These have been extensively studied with respect to various biosorption isotherms, derived from sorption experiments and the effect of various factors, such as pH, biomass pretreatment, and the biomass of the organisms. Precipitation of the excess metal ions, through nucleation reactions, then occurs at the cell surface. All microorganisms have a negative charge on their cell surface due to the presence of anionic structures, which enable them to bind to metal cations. The negatively charged groups that are involved in metal adsorption are the alcohol, amine, carboxyl, ester, hydroxyl, sulfhydryl, phosphoryl, sulfonate, thioether, and thiol groups [53].

An analysis of the cell wall components, which vary among the different microorganisms, helps in assessing metal uptake by different microorganisms. The peptidoglycan layer in Gram-positive bacteria, which contains alanine, glutamic acid, meso-di-aminopimelic acid, polymer of glycerol and teichoic acid, and that of the Gram-negative bacteria, which contains enzymes, glycoproteins, lipopolysaccharides, lipoproteins, and phospholipids, are the active sites involved in metal binding processes [57,59,60]. Metals and metalloids are attached to these ligands on cell surfaces, which displace essential metals from their normal binding sites. Once the metal and metalloid are bound, microbial cells can transform them from one oxidation state to another, thus reducing their toxicity [4]. Gavrilescu [53] reported that the cell walls of bacteria are polyelectrolyte, which interacts with metal ions to maintain electro-neutrality by mechanisms of covalent bonding, extracellular precipitations, redox interactions, and van der Waals forces.

The rigid cell wall of fungi is made up of chitin, inorganic ions, lipids, nitrogen-containing polysaccharide, polyphosphates, and proteins. They can tolerate and detoxify metal ions by active uptake, extracellular and intracellular precipitation, and valence transformation, with many absorbing heavy metals into their mycelium and spores. The surface of their cell wall acts as a ligand for binding metal ions, resulting in the removal of metals [60]. The first barrier includes excreted substances like organic acids or/and proteins with an ability to immobilize heavy metals. The second barrier includes the (unspecific) binding of heavy metals by the cell wall and melanins located in the cell wall. Toxic heavy metals that could not be detained outside the cell must be detoxified inside the cell [61].

The cell wall of all classes of algae is composed of cellulose with sulfonated polysaccharides present in the cell wall of brown and red algae. Other binding sites in algae are polysaccharides such as alginic acid, glycan, mannan, proteins, and xylans. The cell wall of cyanobacteria is composed of peptidoglycan, and some species also produce sheaths and extracellular polymeric substances, which are used for sorption. Characteristics of the biomass, chemical and physical properties of the metal of interest, and pH of the solution, influence the sorption capacity of algae [59].

Non-essential metal uptake usually consists of transporters which are committed to the acquisition of vital organic and inorganic ions. These transporters assist in either the co-transport of these metals in complexes with low-molecular-mass ligands, or in the direct uptake of non-essential metals [62]. Microorganisms can also secrete many kinds of metal-binding metabolites, produce extracellular polymeric substances, which are made up of polysaccharide, capsules, slimes and sheaths, and biofilms, depending on the make-up of the polysaccharide and associated components. Biofilms bind substantial quantities of heavy metals under pristine conditions and serve as a medium for the precipitation of insoluble mineral phases [57].

## 6. Biosorption Capacity of Various Microbial Biosorbents

Various microbial biomass has different biosorptive abilities, which also varies considerably within each group. However, the biosorption capacity of each biosorbent depends on its prehistory and pretreatment, as well as the experimental conditions. The biosorbent should be cheap, effective, and easy to grow and harvest. The organism should also lend itself to alteration of the bioreactor configuration, as well as physical and chemical conditions to enhance biosorption [57].

Bacteria have been used as biosorbents owing to their ubiquity, size, ability to grow under controlled conditions, and resilience to an extensive range of environmental conditions [63,64]. Various heavy metals have been tested on bacteria species such as *Pseudomonas*, *Enterobacter*, *Bacillus*, and *Micrococcus* species (Table 2). Their excellent sorption capacity is due to their high surface-to-volume ratios and their numerous potential active chemosorption sites, such as the teichoic acid on the cell wall [58].

Sinha et al. [65] designed a laboratory scale sequential bioreactor for the removal of mercury from synthetic effluent (10 mg/L of Hg). The efficiency of mercury removal by *Bacillus cereus* (immobilize on alginate) was 104.1 mg/g on the third day. *Micrococcus luteus* was used to remove a large amount of Pb from a synthetic medium. Under optimal conditions, the removal capacity was 1965 mg/g [66]. Kim et al. [67] also designed a batch system using zeolite-immobilized *Desulfovibrio desulfuricans* for Cu, Cr, and Ni removal from contaminated seawater (Table 2). The removal efficiency was 98.2, 99.8, and 90.1 mg/g, respectively, after about seven days.

Yeasts and molds are easy to cultivate, can be genetically and morphologically manipulated, and can produce a high biomass yield. They are widely used in a variety of large-scale industrial fermentation processes, producing ferrichrome, gallic and kojic acid, and enzymes like lipases, glucose isomerase, pectinases, amylases, and glucanases [63]. They are extensively used as biosorbents for the removal of toxic metals from polluted wastewaters, with excellent abilities for metal uptake and recovery [68,69,70]. They have developed a complex defense system to neutralize heavy metal toxicity. Akar et al. [69] evaluated the Pb removal potential of *Botrytis cinerea* in a batch reactor. Lead(II) ions were found to be extracellularly accumulated on the cell surface and the rate of accumulation was affected by the pH, contact time, and initial metal concentration. The sorption capacity of Pb by *B. cinerea* was found to be 107.1 mg/g at an initial Pb concentration of 350 mg/L, after 180 min. Fu et al. [70] recently reported the biosorption of Cu(II) ions by mycelial pellets of *Rhizopus oryzae*. The effects of pellet diameter, solution pH, contact time, initial metal concentration, and temperature were evaluated. Metal removal efficiency of Cu(II) ions using mycelial pellets was observed to be 34 mg/g after two hours (Table 2). Sharma and Adholeya [71] reported that *Paecilomyces lilacinus* fungi accumulate only 24% of chromium from spent chrome effluent supplemented with cane sugar, while 100% removal was observed from a synthetic medium. Srivastava and Thakur [72] also reported the efficiency of chromium removal by *Aspergillus* sp. from tannery effluent. Eighty-five percent of the chromium was removed at pH 6 in a bioreactor system from the synthetic medium, compared to a 65% removal from the tannery effluent. This is because of the presence of organic pollutants that inhibit the growth of the fungal species.

Algae have also been used as biosorbents for heavy metal removal. Brown algae have gained prominence as good biosorbents because of their high sorption capacity. Red, green, and brown algae have been used for adsorption studies and are all readily available in marine and fresh water environments [64]. Algae are autotrophic, thus require a low number of nutrients and produce a large biomass compared to other microbial biosorbents. They have a high sorption capacity and are readily available in large quantities [7]. The sorption capacity of six different algae were evaluated for the recovery of Cd, Cu, Ni, Pb, and Zn from an aqueous solution by Romera et al. [55]. The maximum sorption of Cd (32.3 mg/g), Pb (63.7 mg/g), and Zn (21.6 mg/g), were recorded for *Asparagopsis armata*, while the maximum Cd (21.8 mg/g), Pb (63.3 mg/g), and Zn (23.8 mg/g) uptake, occurred in *Codium vermilara* (Table 2). Algae are effective biosorbents for the removal of Sb(III) from aqueous solutions [73]. The maximum adsorption capacity of Sb(III) by the algae *Sargassum muticum* was 5.5 mg/g, at pH 5. A slight effect of pH was observed in the removal efficiency of Sb by *S. muticum*.

## 7. Conclusions

This review revealed the contributions of the various biosorbents which are potentially effective and readily available for heavy metal removal. These biosorbents present attractive opportunities as low cost means of protecting the environment from pollution. Biosorbent selection and implementation for industrial wastewater management and soil remediation requires more effort, as most reported adsorption studies have been confined to laboratory investigations in a batch system. A sustainable approach needs to be developed in order to select the most appropriate biosorbent, operating conditions, and efficient mechanism of heavy metal removal in industrial effluent, to sufficiently address the major challenges involved. Also, in order to develop a reliable biosorption process, more research is needed in biosorbent characterization, in terms of surface morphology and area, zeta potential, functional groups, and particle size, as these are important in biosorption experiments, influenced by the pretreatment of the biosorbents. Equally, growing microbial biomass with the potential for metal uptake needs further investigation, with the aim of exploring the metabolic potential of these growing biomass and their application in industrial wastewater management.

## Figures and Tables

**Table 1:** Toxicity of heavy metals to life forms.

Metal	Source	Effects on Human	Efeects on Plants	Effects on Microrganisms	Reference
Antimony	Coal combustion, mining, smelting, soil erosion, volcanic eruption	Cancer, cardiovascular diseases, conjunctivitis, dermatitis, liver diseases, nasal ulceration, respiratory diseases	Decreases synthesis of some metabolites, growth inhibition, inhibit chlorophyll synthesis	Inhibit enzyme activities, reduced growth rate	[18,19]
Arsenic	Atmospheric deposition, mining, pesticides, rock sedimentation, smelting	Brain damage, cardiovascular and respiratory disorder, conjunctivitis, dermatitis, skin cancer	Damage cell membrane, inhibition of growth, inhibits roots extension and proliferation, interferes with critical metabolic processes, loss of fertility, yield and fruit production, oxidative stress, physiological disorders	Deactivation of enzymes	[20,21,22]
Beryllium	Coal and oil combustion, volcanic dust	Allergic reactions, berylliosis, cancer, heart diseases, lung diseases	Inhibits seed germination	Chromosomal aberration, mutation	[18,23]
Cadmium	Fertilizer, mining, pesticide, plastic, refining, welding	Bone disease, coughing, emphysema, headache, hypertension, itai-itai, kidney diseases, lung and prostate cancer, lymphocytosis, microcytic hypochromic anemia, testicular atrophy, vomiting	Chlorosis, decrease in plant nutrient content, growth inhibition, reduced seed germination	Damage nucleic acid, denature protein, inhibit cell division and transcription, inhibits carbon and nitrogen mineralization	[5,24,25,26,27]
Chromium	Dyeing, electroplating, paints production, steel fabrication, tanning, textile	Bronchopneumonia, chronic bronchitis, diarrhea, emphysema, headache, irritation of the skin, itching of respiratory tract, liver diseases, lung cancer, nausea, renal failure, reproductive toxicity, vomiting	Chlorosis, delayed, senescence, wilting, biochemical lesions, reduced biosynthesis germination, stunted growth, oxidative stress	Elongation of lag phase, growth inhibition, inhibition of oxygen uptake	[28,29,30]
Copper	Copper polishing, mining, paint, plating, printing operations	Abdominal pain, anemia, diarrhea, headache, liver and kidney damage, metabolic disorders, nausea, vomiting	Chlorosis, oxidative stress, retard growth	Disrupt cellular function, inhibit enzyme activities	[2,5,24,31]
Mercury	Batteries, coal combustion, geothermal activities, mining, paint industries, paper industry, volcanic eruption, weathering of rocks	Ataxia, attention deficit, blindness, deafness, decrease rate of fertility, dementia, dizziness, dysphasia, gastrointestinal irritation, gingivitis, kidney problem, loss of memory, pulmonary edema, reduced immunity, sclerosis	Affects antioxidative system, affects photosynthesis, enhance lipid peroxidation, induced genotoxic effect, inhibit plant growth, yield, nutrient uptake and homeostasis, oxidative stress	Decrease population size, denature protein, disrupt cell membrane, inhibits enzyme function	[24,32,33]
Lead	Coal combustion, electroplating, manufacturing of batteries, mining, paint, pigments	Anorexia, chronic nephropathy, damage to neurons, high blood pressure, hyperactivity, insomnia, learning deficits, reduced fertility, renal system damage, risk factor for Alzheimer’s disease, shortened attention span	Affects photosynthesis and growth, chlorosis, inhibit enzyme activities and seed germination, oxidative stress	Denatures nucleic acid and protein, inhibits enzymes activities and transcription	[5,24,34,35]
Nickel	Electroplating, non-ferrous metal, paints, porcelain enameling	Cardiovascular diseases, chest pain, dermatitis, dizziness, dry cough and shortness of breath, headache, kidney diseases, lung and nasal cancer, nausea	Decrease chlorophyll content, inhibit enzyme activities and growth, reduced nutrient uptake	Disrupt cell membrane, inhibit enzyme activities, oxidative stress	[24,25,36]
Selenium	Coal combustion, mining	Dysfunction of the endocrine system, gastrointestinal disturbances, impairment of natural killer cells activity, liver damage	Alteration of protein properties, reduction of plant biomass	Inhibits growth rate	[2,37]
Silver	Battery manufacture, mining, photographic processing, smelting	Argyria and argyrosis, bronchitis, cytopathological effects in fibroblast and keratinocytes, emphysema, knotting of cartilage, mental fatigue, nose, throat and chest irritation, rheumatism	Affects homeostasis, decrease chlorophyll content, inhibits growth	Cell lysis, inhibit cell transduction and growth	[38,39]
Thallium	Cement production, combustion of fossil fuels, metal smelting, oil refining	Alopecia, ataxia, burning feet syndrome, coma, convulsions, delirium, fatigue, gastroenteritis, hair fall, hallucinations, headache, hypotension, insomnia, nausea, tachycardia, vomiting	Inhibits enzyme activities, reduced growth	Damages DNA, inhibits enzyme activities and growth	[18,40]
Zinc	Brass manufacturing, mining, oil refinery, plumbing	Ataxia, depression, gastrointestinal irritation, hematuria, icterus, impotence, kidney and liver failure, lethargy, macular degeneration, metal fume fever, prostate cancer, seizures, vomiting	Affects photosynthesis, inhibits growth rate, reduced chlorophyll content, germination rate and plant biomass	Death, decrease in biomass, inhibits growth	[25,41]

**Table 2:** Metal biosorption by different microbial biosorbents.

Microbial Group	Microbial Biosorbent	Metal	pH	Temperature (°C)	Time (h)	Initial Metal Ion Concentration (mg/L)	Sorption Capacity (mg/g)	Reference
Bacteria	*Bacillus cereus* (Immobilize on alginate)	Hg	7	30	72	10	104.1	[65]
	*B. laterosporus*	Cd	7	25	2	1000	159.5	[74]
		Cr(VI)	2.5				72.6	
	*B. licheniformis*	Cd	7	25	2	1000	142.7	
		Cr(VI)	2.5				62	
	*Desulfovibrio desulfuricans* (immobilize on zeolite)	Cu	7.8	37	168	100	98.2	[67]
		Ni				100	90.1	
		Cr(VI)				100	99.8	
	*Enterobacter cloacae*	Pb	-	30	48	7.2	2.3	[75]
	*Kocuria rhizophila*	Cd	8	35	1	150	9.07	[76]
		Cr	4			150	14.4	
	*Micrococcus luteus*	Cu	7	27	12	80.24	408	[66]
		Pb				272.39	1965	
	*Pseudomonas aeruginosa*	Co	5.2	25	10	58.93	8.92	[77]
		Ni	5.5			58.69	8.26	
		Cr(III)	3.4			52	6.42	
	*P. jessenii*	Ni	-	25	6	275	1.36	[78]
		Cu				300	10.22	
		Zn				400	4.39	
	*Pseudomonas* sp.	Ni		25	6	275	2.79	
		Cu				300	5.52	
		Zn				275	3.66	
	Sulphate-reducing bacteria	As(III)	6.9	-	24	1	0.07	[79]
		As(V)					0.11	
Fungi	*Aspergillus niger*	Cu	5	30	1	100	15.6	[68]
		Pb	4.5			100	34.4	
		Cr(VI)	3.5			50	6.6	
	*Botrytis cinereal*	Pb	4	25	1.5	350	107.1	[69]
	*Phanerochaete chrysosporium* (immobilized on loofa sponge)	Pb	6	20	1	100	88.16	[80]
		Cu				100	68.73	
		Zn				100	39.62	
	*Pleurotus platypus*	Ag	6	20	2	200	46.7	[81]
	*Rhizopus oryzae*	Cu	4	35	2	100	34	[70]
Algae	*Asparagopsis armata*	Cd	6	-	2	135	32.3	[55]
		Ni	6			141	17.7	
		Zn	6			182	21.6	
		Cu	5			134.4	21.3	
		Pb	4			124	63.7	
	*Codium vermilara*	Cd	6	-	2	135	21.8	[55]
		Ni	6			147	13.2	
		Zn	6			182	23.8	
		Cu	5			140	16.9	
		Pb	5			83	63.3	
	*Cystoseira barbata*	Cd	4	20	1	117.4	37.6	[82]
		Ni				224.8	78.7	
		Pb				414	196.7	
	*Lessonia nigrescens*	Ar(V)	2.5	20	5	200	45.2	[83]
	*Sargassum muticum*	Sb	5	23	4	10	5.5	[73]
	*Spirogyra* sp.	Pb	5	25	1.6	200	140	[84]
